# A multilevel analysis of factors associated with vitamin A supplementation among children aged 6–35 months in Ethiopia

**DOI:** 10.3389/fpubh.2023.1052016

**Published:** 2023-02-23

**Authors:** Tsegaw Amare, Tseganesh Sime, Gebrehiwot Lema Legese, Menberesibhat Getie Ferede, Melaku Birhanu Alemu

**Affiliations:** ^1^Department of Health Systems and Policy, Institute of Public Health, College of Medicine and Health Science, University of Gondar, Gondar, Ethiopia; ^2^Maternal and Child Health Unit, University of Gondar Comprehensive Specialized Hospital, Gondar, Ethiopia; ^3^Department of Internal Medicine, School of Medicine, College of Medicine and Health Science, University of Gondar, Gondar, Ethiopia; ^4^Department of Anatomy, School of Medicine, College of Medicine and Health Science, University of Gondar, Gondar, Ethiopia

**Keywords:** vitamin A supplementation, Ethiopia, multilevel analysis, childhood illness, vitamin A deficiency (VAD)

## Abstract

**Background:**

Vitamin A deficiency is among the leading preventable causes of childhood morbidity and mortality that might be attributable to the low uptake of vitamin A supplementation (VAS). Factors contributing to its low utilization are not researched at the national level and with the appropriate model. Therefore, this study aimed at identifying the magnitude and the individual- and community-level factors associated with vitamin A supplementation among children aged 6–35 months in Ethiopia.

**Methods:**

We have used the Ethiopian mini demographic and health survey data, which was conducted from 21 March to 28 June 2019. A weighted sum of 2,362 mothers having children aged 6–35 was extracted. Considering the hierarchical nature of the data, we fitted the multilevel multivariable logistic regression model. Adjusted odds ratio (AOR) with a 95% confidence interval (CI) was reported and variables with a *p*-value of < 0.05 were declared to be significantly associated factors.

**Results:**

In this study, 43.4% (95% CI: 41.4–45.4%) of children have taken the VAS. Moreover, the 12–23 age of the child (AOR = 2.64; 95% CI: 1.88–3.72), 30–34 age of the mother (AOR = 3.34; 95% CI: 1.21–9.20), middle household wealth status (AOR = 1.75; 95% CI: 1.06–2.90), and four and above antenatal care (AOR = 2.90; 95% CI: 1.90–4.43) are the individual-level factors associated with VAS whereas being from Amhara (AOR = 2.20; 95% CI: 1.29–3.76) and Tigray (AOR = 2.16; 95% CI: 1.17–3.98) regions is a community-level factor significantly associated with the uptake of VAS.

**Conclusion:**

Overall, a low proportion of children have taken the VAS in Ethiopia. The higher age of the child and mother, full antenatal care, and improved wealth status positively influence VAS. Moreover, a child from the Tigray or Amhara regions was more likely to get VAS. Therefore, an intervention has to be designed to address the VAS uptake among young mothers, and working to improve the wealth status of the household would be helpful. Moreover, the advocacy of antenatal care and minimizing the regional disparity through encouraging the uptake in the rest of the regions would help increase the national-level uptake of VAS.

## Background

Vitamin A deficiency (VAD), the leading preventable contributor to childhood blindness, diarrhea, and measles infection (1, 2), is a disorder that occurs when our body is unable to satisfy the physiological needs due to the inadequate body storage of vitamin A (3, 4).

Even though VAD is decreasing worldwide, its prevalence in Sub-Saharan Africa (SSA; 48%) and South Asia (44%) is alarmingly high in children (5). In 2013, nearly 94,500 and 11,200 deaths from childhood diarrhea and measles infection were attributable to VAD (5). The incidence of deaths was also skewed to the developing countries. For instance, SSA and South Asia account 95% of deaths due to VAD (5). In Ethiopia, 37.7% of children had deficient clinical serum vitamin A levels (6).

Hence, one of the strategies for treating VAD is vitamin A supplementation (VAS) during a childhood period (7, 8). According to the World Health Organization (WHO) recommendation, a 100,000 international unit (IU) dose of vitamin A supplementation should be given for 6–11 months and 200,000 IU for 12–59 months aged children every 4–6 months who are living in affected areas (9). Moreover, the regular uptake of the recommended dose of vitamin A supplementation decreases childhood mortality by 12−28% (10, 11).

However, the coverage of VAS is not in line with its public health importance. In 2020, there was a 60% unmet demand for VAS worldwide (12). Moreover, the VAS coverage in SSA was 65% in 2018 (13) and it ranges from 40.8% in Guinea to 88.4% in Senegal (14).

In Ethiopia, VAS is given to children aged 6–59 two times a year as part of the expanded program of immunization (EPI) for the last two decades (15, 16). The country implemented a variety of strategies such as routine health extension programs, enhanced outreach strategies, and community health day modalities to address the unmet demand for VAS (17). The routine VAS has saved 167,563 to 376,030 child lives between the years 2005 and 2019 (18). However, more than half (55%) of the eligible children were not supplemented in 2016 (19) and 43% of children were in demand of VAS in 2019 (20). It is below the 95% country's target of VAS in 2020 (21). On the other hand, the country has planned to strengthen and scale up VAS to children by 2025 (22).

According to different studies, different factors were associated with the uptake of VAS. Among them, sex and age of the child, mother's age, maternal occupation, maternal education, households' wealth status, possessing a television, residence, region, knowledge, information provider, antenatal care, place of delivery, and postnatal check-up were the determinants of the uptake of VAS (14, 23–30).

Despite the presence of evidence on identifying the factors contributing to the uptake of VAS in Ethiopia, the available studies were not identified factors at the national level (26) and the national-level studies were not conducted with the appropriate model that considers the hierarchical nature of the national survey. Hence, in the multilevel model (random effect model), unlike the fixed effects model (the conventional logistic analysis), the effects of group-level predictors will not be confounded and the effects of different level (individual and community level) variables can be estimated. The methodological issues of multilevel analysis are depicted elsewhere (31). In addition, some of the available evidence is not up-to-date to explore the individual- and community-level factors for the uptake of VAS (29, 30).

Therefore, this study aimed at identifying the factors associated with the uptake of VAS in Ethiopia using the most recent Ethiopian demographic and health survey conducted in 2019 and employing the multilevel analysis to account for the hierarchical nature of the survey. Thus, the finding of this study will help to make evidence-based decisions by having updated information on the country's national-level VAS and its determinants.

## Methods and materials

### Study settings

Ethiopia is the second most populous country in Africa, with a total population of near 122 million according to the 2022 United Nations data (32). Based on the World Bank report, Ethiopia is one of the world's poorest countries, with a per capita income of US$944 in 2021 (33). Currently, a three-tier healthcare delivery system is being implemented in Ethiopia. Primary-level health care includes health posts, health centers, and primary hospitals, secondary-level health care delivered by the general hospitals, and tertiary-level health care carried by specialized hospitals (34).

### Data source, sampling procedure, and population

The 2019 Ethiopian Mini Demographic and Health Survey (EMDHS) was the data source for this study. The cross-sectional survey was conducted from 21 March to 28 June 2019, using a complete list of 149,093 enumeration areas (EAs) as a sampling frame. The survey was conducted by a two-stage stratified sampling. That is each region was stratified into urban and rural areas to yield 21 sampling strata. First, a total of 93 urban EAs and 212 rural EAs were chosen with a probability proportionate to EA size. Then, in the second stage of selection, a fixed number of 30 households per cluster were selected with an equal probability of systematic selection from the newly created household listing, after a household listing operation was carried out in all selected EAs from January to April 2019 (the resulting lists of households served as a sampling frame for the selection of households in the second stage) (20). In this study, we used the birth record dataset (BR data). From a total of 2,403 mothers having children aged 6–35 months, 2,362 weighted samples were included for analysis. Sample weighting was conducted using the “*Svy*” command.

### Study variables

The outcome variable of this study was vitamin A supplementation among children aged 6–35 in the last 6 months (*yes/no*). The independent variables were individual-level variables such as the age of the child, age of the mother, sex of the child, sex of household head, religion, marital status, mother's education, husband's education, wealth status, working status, birth order, parity, possession of radio, possession of a television, number of under-five children in the household, place of delivery of the child, mothers having ANC, and mothers having postnatal care. The community-level variables were residence and region. A region in this study was classified into five Oromia, Amhara, Southern Nation Nationalities and Peoples Region (SNNPR), Tigray, and others. The ‘others' region contains Addis Ababa, Somalia, Afar, Dire Dawa, Benishangul, and Gambella due to their low number of eligible participants for this study. The questionnaire of the survey was pretested and 2 days of training were given to the data collectors and supervisors before the onset of actual data collection.

### Data management and analysis

Stata version 14 was used for data management (extraction, re-coding, and categorization) and statistical analysis (identifying the factors contributing to VAS). Considering the hierarchical nature of the data, a multilevel multivariable logistic analysis was employed for assessing the factors associated with VAS. While conducting multilevel analysis, four models were fitted, namely, the null model (a model without explanatory variables), model I (a model with individual-level explanatory variables only), model II (a model with community-level variables only), and model III (a model with both individual- and community-level variables). Both bivariable and multivariable multilevel analyses were used. Variables with a *p*-value of <0.20, in the bivariable analysis, were eligible for the multivariable analysis. In the multivariable analysis, an adjusted odds ratio (AOR) with a 95% confidence interval (CI) was reported and variables with a *p*-value of <0.05 were declared to be a significantly associated factor. For assessing the cluster-level variability of VAS, we have employed the random effect analysis calculating the Intraclass Correlation Coefficient (ICC) and Proportional Change in Variance (PCV). Finally, model fitness was checked using Deviance.

### Ethical considerations

We have used the secondary data from the EDHS, which was conducted under the Declaration of Helsinki. Through online request, we accessed the dataset from the DHS website (https://dhsprogram.com) and personal identifiers were not available on the dataset. Since we have used publicly accessible data, ethical approval was not needed.

## Results

### Background characteristics of the respondents

In this study, 2,362 mothers having a child aged 6–35 months were included. The mean (± standard deviation) age of the mothers and children was 28.13 ± 6.42 years and 19.16 ± 8.29 months, respectively. Among eligible children, only 43.4% (95% CI: 41.4–45.4%) have taken the VAS. The majority 391 (47.07%) of the children who have taken the supplementation were aged between 24 and 35 months and 279 (54.55%) of the children who have supplemented with vitamin A were from the Amhara region (Table 1).

**Table 1:** Background characteristics of the respondents having children aged 6–35 months in Ethiopia (*N* = 2,362).

**Variables**	**VAS non-uptake**		**VAS uptake**	
	**Frequency**	**Percent**	**Frequency**	**Percent**
**Sex of household head**				
Male	1,165	56.59	894	43.41
Female	180	59.36	123	40.64
**Age of the mother (years)**				
15–19	109	71.17	42	28.83
20–24	305	60.49	199	39.51
25–29	455	59.02	316	40.98
30–34	237	50.56	232	49.44
35–39	158	52.06	142	47.94
40–49	91	51.12	87	48.88
**Sex of the child**				
Male	713	57.07	536	42.93
Female	932	56.80	481	43.20
**Age of the child (months)**				
6–11	355	70.58	148	29.42
12–23	551	53.53	478	46.47
24–35	439	52.93	391	47.07
**Highest educational level**				
No education	694	59.91	464	40.09
Primary	500	55.71	398	44.29
Secondary and higher	151	49.37	155	50.63
**Current marital status**				
Unmarried	66	48.09	71	51.91
Married	1,279	57.49	946	42.51
**Religion**				
Orthodox	406	48.31	435	51.69
Muslim	480	60.57	313	39.43
Protestant	420	61.63	261	38.37
Other^*^	39	82.43	8	17.57
**Wealth index**				
Poorest	321	65.13	172	34.87
Poorer	320	62.95	188	37.05
Middle	212	45.99	249	54.01
Richer	244	59.94	163	40.06
Richest	249	50.35	245	49.65
**Household has television**				
No	1,165	58.79	817	41.21
Yes	180	47.35	200	52.65
**Household has radio**				
No	996	56.71	761	43.29
Yes	349	57.61	256	42.39
**Type of place of residence**				
Urban	334	52.33	305	47.67
Rural	1,011	58.66	712	41.34
**Region**				
Oromia	531	58.28	380	41.72
Amhara	233	45.45	279	54.55
SNNPR	313	66.10	161	33.90
Tigray	65	41.24	92	58.76
Others^**^	204	65.95	105	34.05

* Catholic and traditional.

** Addis Ababa, Somalia, Afar, Dire Dawa, Benishangul, Gambella, and Harari.

### Maternity-related characteristics of the respondents

In this study, 236 (44.05%) of children whose mother is nulliparous have taken the vitamin A supplementation, whereas 529 (52.26%) of children whose mothers had four and above antenatal care have taken the supplementation. A total of 1,194 (59.01%) children whose mothers have not had a postnatal check-up within the last 2 months have not taken the VAS (Table 2).

**Table 2:** Maternity-related characteristics of the respondents having children aged 6–35 months in Ethiopia (*N* = 2,362).

**Variables**	**VAS non-uptake**		**VAS uptake**	
	**Frequency**	**Percent**	**Frequency**	**Percent**
**Parity**				
Null parity	230	55.95	236	44.05
Multiparity	611	57.58	450	42.42
Grand multiparity	434	56.75	331	43.25
**Number of ANC follow-up**				
No visit	423	70.18	180	29.82
One visit	53	63.02	31	36.98
Two visits	116	64.77	63	35.23
Three visits	270	55.79	214	44.21
Four and above visits	483	47.74	529	52.26
**Place of delivery**				
Home	703	62.25	427	37.75
Health facility	641	52.08	590	47.92
**Having postnatal check-up within 2 months**				
No	1,194	59.01	829	40.99
Yes	151	44.59	188	55.41
**Number of children 5 and under in the household**				
0–1	614	54.15	520	45.85
2–3	712	59.22	491	40.78
4–5	19	73.28	6	26.72

### Multilevel logistic regression analysis

In this study, the ICC of the null model indicated that 25.4% of the variability of VAS is attributable to the differences between the clusters. Moreover, 27.7% of the variability of VAS was explained by both individual- and community-level variables. Furthermore, model III was the best-fitted model since it had the lowest deviance.

In a multilevel binary logistic regression, the age of the mother, age of the child, wealth index, the household that has television, type of place of residence, region, the number of ANC follow-ups, place of delivery, having postnatal checkups within 2 months, the number of under-five children in the household, type of residence, and region were associated with VAS with a *p*-value of <0.2. In the multivariable logistic analysis model, the age of the mother, the age of the child, wealth index, ANC follow-up, and region were significantly associated with VAS with a *p*-value of < 0.05.

A child whose mother aged 30–34, 35–39, and 40–49 had 3.34 (AOR = 3.34; 95% CI: 1.21–9.20), 3.06 (AOR = 3.06; 95% CI: 1.00–9.34), and 2.62 (AOR = 2.62; 95% CI: 1.02–6.70) times higher odds of getting VAS than a child whose mother's age was between 15 and 19, respectively. Similarly, a child with the age range of 12–23 and 24–35 months had 2.64 (AOR = 2.64; 95% CI: 1.88–3.72) and 2.20 (AOR = 2.20; 95% CI: 1.37–3.56) times higher odds of VAS than a child with an age range of 6–11 months. Moreover, a child from a middlingly wealthy family was 1.75 (AOR = 1.75; 95% CI: 1.06–2.90) times more likely to get a VAS as compared to a child from the poorest family. In addition, having three ANC follow-ups and four and above ANC follow-up increases the odds of VAS by 1.91 (AOR = 1.91; 95% CI: 1.05–3.45) and 2.90 (AOR = 2.90; 95% CI: 1.90–4.43) times as compared to no ANC follow-up. On the other hand, a child residing in Amhara and Tigray would increase the odds of VAS by 2.20 (AOR = 2.20; 95% CI: 1.29–3.76) and 2.16 (AOR = 2.16; 95% CI: 1.17–3.98) times as compared to other regions (Afar, Somalia, Dire Dawa, Addis Ababa, Harari, Benishangul, and Gambella; Table 3).

**Table 3:** Multilevel multivariable logistic regression output for factors associated with vitamin A supplementation in Ethiopia.

**Variables**	**Null model**	**Model I AOR (95% CI)**	**Model II AOR (95% CI)**	**Model III AOR (95% CI)**
**Age of the mother (years)**				
15–19		1		1
20–24		2.06 (0.85–4.99)		2.03 (0.84–4.97)
25–29		2.42 (0.85–5.91)		2.22 (0.84–5.88)
30–34		3.46 (1.27–9.46)^*^		3.34 (1.21–9.20)^*^
35–39		3.13 (1.03–9.46)^*^		3.06 (1.00–9.34)^*^
40–49		2.72 (1.06–6.95)^*^		2.62 (1.02–6.70)^*^
**Age of the child (months)**				
6–11		1		1
12–23		2.65 (1.89–3.71)^**^		2.64 (1.88–3.72)^***^
24–35		2.19 (1.36–3.52)^*^		2.20 (1.37–3.56)^**^
**Wealth index**				
Poorest		1		1
Poorer		0.91 (0.62–1.33)		0.88 (0.60–1.30)
Middle		1.80 (1.10–2.97)^*^		1.75 (1.06–2.90)^*^
Richer		0.91 (0.51–1.60)		0.88 (0.49–1.58)
Richest		0.79 (0.33–1.88)		0.69 (0.27–1.77)
**The household has a television**				
No		1		1
Yes		1.20 (0.55–2.61)		1.14 (0.53–2.49)
**Number of under 5 children in the household**				
0–1		1		1
2–3		0.83 (0.61–1.12)		0.85 (0.63–1.15)
4–5		0.52 (0.06–4.86)		0.59 (0.07–5.10)
**Place of delivery**				
Home		1		1
Health facility		1.22 (0.75–1.95)		1.20 (0.74–1.94)
**Number of ANC follow-up**				
No visit		1		1
One visit		1.60 (0.73–3.47)		1.48 (0.68–3.22)
Two visits		1.34 (0.72–2.49)		1.27 (0.68–2.37)
Three visits		2.00 (1.10–3.62)^*^		1.91 (1.05–3.45)^*^
Four and above visits		3.09 (2.03–4.68)^***^		2.90 (1.90–4.43)^***^
**Having postnatal check-up within 2 months**				
No		1		1
Yes		1.35 (0.87–2.10)		1.37 (0.88–2.13)
**Type of place of residence**				
Urban			1	1
Rural			0.58 (0.38–0.88) ^*^	0.68 (0.35–1.33)
**Region**				
Oromia			1.65 (0.96–2.83)	1.36 (0.75–2.47)
Amhara			2.96 (1.78–4.93)^*^	2.20 (1.29–3.76)^**^
SNNPR			1.03 (0.67–1.58)	0.84 (0.52–1.36)
Tigray			3.21 (1.84–5.62)^*^	2.16 (1.17–3.98)^*^
Others^*$*^			1	1

* p < 0.05.

** p < 0.01.

*** p < 0.001. ^*$*^Addis Ababa, Somalia, Afar, Dire Dawa, Benishangul, and Gambella.

## Discussion

This study was designed to identify the individual- and community-level factors, which affect the uptake of VAS in Ethiopia. Maternal and child age, wealth index, ANC follow-up, and region were among the factors, which affect the VAS of the child significantly.

In this study, 43.4% (41.4%−45.4%) of children have taken VAS, which is in line with another similar study conducted in Ethiopia which showed that the VAS in Ethiopia was 44.4% (35). But the finding of this study is higher than another study conducted that indicated that the uptake of vitamin A-rich food in Ethiopia was 38.99% (36). This might be used as an indicator that food-enriched uptake of vitamin A is falling behind the supplementation. Hence, action has to be taken to enhance the uptake of vitamin A-rich food hand in hand with supplementation. The trend on VAS has not shown a significant change from the 45% of VAS in 2016 (19). However, it is lower than the 59.4% coverage of VAS in SSA countries (25) and the 65% average proportion of VAS for lower-income countries (37). The difference might be due to the later studies including children of 6–59 months whereas this study included children of 6–35 months. On the one hand, this finding also underlines that Ethiopian progress is distant even from its peer SSA and low-income countries.

Moreover, this study identified important individual- and community-level factors. Accordingly, the older maternal and child age was significantly associated with the fewer odds of VAS. A child whose mother was aged 30–34, 35–39, and 40–49 had 3.34, 3.06, and 2.62 times higher odds of getting VAS than a child whose mother's age was between 15 and 19, respectively. The finding has supported another study conducted in Ethiopia and elsewhere in SSA (14, 38). The variation could be associated with a lack of awareness and paying less attention among young mothers (30, 39). This might become a huge loss when we consider that teenage pregnancy is common in Ethiopia (40). Therefore, health education programs on the importance of VAS shall target younger mothers to increase the uptake of VAS. Similarly, a child with the age range of 12–23 and 24–35 months had 2.64 and 2.20 times higher odds of getting a VAS than a child in the age range of 6–11 months. This might be related to the culture of Ethiopia where after delivery a mother exposes herself late during the postpartum period. A study conducted somewhere showed a different association (14, 39). Nevertheless, the health extension workers and the women development armies should pay attention to the good uptake of VAS among lower-aged children. Moreover, a child from a middle-wealth family had 1.75 times more likely to get a VAS as compared to a child from the poorest family. This is similar to the other study conducted in Ethiopia (35). Even though VAS is given in Ethiopia free of charge, associated indirect costs might be hindering the poorest households from getting the supplementation.

Consistent with other studies conducted in Ethiopia (30, 35), the odds of having three and four and above ANC follow-up increases the odds of VAS by 1.91 and 2.90 compared with no ANC follow-up. This is supported by antenatal contact, which is usually the entry point for a mother for the subsequent maternal–child health service utilization including the VAS, which leads to a high level of uptake. This is because, during antenatal care, the health professional is usually aware and consults the pregnant woman on the care of the child and herself. Therefore, we also underline that inclusive maternal and child health service awareness creation including the VAS should be strengthened during antenatal contact.

Furthermore, a child who was in Amhara and Tigray would increase the odds of VAS by 2.20 and 2.16 times as compared to other regions (Afar, Somalia, Dire Dawa, Addis Ababa, Harari, Benishangul, and Gambella). The socio-demographic and cultural variation between regions might be contributed to the uptake variation in the regions. A spatial analysis conducted in Ethiopia also indicated the need for immediate interventions in Afar, Somali, and some pocket areas in Addis Ababa for the uptake of vitamin A (30). Moreover, interventions such as promoting VAS in other regions should be considered to achieve the national target of VAS.

Even though the study used the national-level data and fitted the appropriate model for the nature of the data (multilevel model), the survey dataset has limitations on having observations for children aged 37–59 months for VAS. Therefore, factors affecting VAS in the above age group were not able to be identified in this research. In addition, due to the use of EMDHS, the absence of important facility and behavioral variables, such as distance of household from the health facility, knowledge of maternal and husband toward the importance of VAS, and household information about the schedule of VAS, might limit the findings of this study.

## Conclusion

Overall, the proportion of VAS among children in Ethiopia is low. The higher age of the child and mother, full antenatal care, and improved wealth status was positively associated with VAS. Moreover, a child from other than Tigray and Amhara regions was less likely to recieve VAS. Therefore, the intervention has to be designed to improve the uptake of VAS among young mothers as most of the country's population is young and teenage pregnancy is common in our country special attention should be paid by the health extension workers and women's health development army to improve the initiate VAS to children in the postpartum period as soon as possible. Working to improve the wealth status of the household would also have a spillover effect on the uptake of VAS. Moreover, the advocacy of antenatal care and minimizing the regional disparity through encouraging the uptake in the rest of the regions would help increase the national-level uptake of VAS.

## Data availability statement

Publicly available datasets were analyzed in this study. This data can be found at: https://dhsprogram.com.

## Author contributions

TA, TS, GL, MF, and MA developed the concept, reviewed the literature, carried out the statistical analysis, interpret and discussed the results, and drafted the manuscript. All authors reviewed the drafted manuscript and approved the submission of the final manuscript.

## References

[1] WHO. Nutrition Landscape Information System (NLiS): Vitamin A deficiency. Geneva: World Health Organization (2009). Available online at: https://www.who.int/data/nutrition/nlis/info/vitamin-a-deficiency#:~:text=Deficiency%20of%20vitamin%20A%20is,outcomes%20of%20pregnancy%20and%20lactation (accessed July 10, 2022).

[2] Semba RD Smith JC. Vitamin A and the immune system. In:Delves PJ, editor. Encyclopedia of Immunology, 2nd ed. Oxford: Elsevier (1998), p. 2488–9. 10.1006/rwei.1999.0627

[3] World Health Organization. Vitamin A deficiency 2009. Available online at: https://www.who.int/data/nutrition/nlis/info/vitamin-a-deficiency (accessed July 10, 2022).

[4] Ross AC Hodges JK Wei C-h Li Y. Vitamin A. In:Prasad AS Brewer GJ, editors. Essential and Toxic Trace Elements and Vitamins in Human Health. Cambridge, MA: Academic Press (2020), p. 202–14. 10.1016/B978-0-12-805378-2.00016-4

[5] Stevens GA Bennett JE Hennocq Q Lu Y De-Regil LM Rogers L. Trends and mortality effects of vitamin A deficiency in children in 138 low-income and middle-income countries between 1991 and 2013: a pooled analysis of population-based surveys. Lancet Glob Health. (2015) 3:e528–e36. 10.1016/S2214-109X(15)00039-X 26275329

[6] Demissie T Ali A Mekonen Y Haider J Umeta M. Magnitude and distribution of vitamin A deficiency in Ethiopia. Food Nutr Bull. (2010) 31:234–41. 10.1177/156482651003100206 20707229

[7] Wedner SH Ross DA. Vitamin A deficiency and its prevention. In:Heggenhougen HK, editor. International Encyclopedia of Public Health. Oxford: Academic Press (2008), p. 526–32. 10.1016/B978-012373960-5.00642-0

[8] World Vision. Nutrition: Vitamin A Supplementation. (2005). Available online at: https://www.wvi.org/nutrition/vitamin-supplementation#:~:text=Vitamin%20A%20supplementation%20is%20a,is%20required%20for%20young%20children (accessed July 10, 2022).

[9] World Health Organization. Children 6-59 Months Receiving Vitamin A Supplements. (2018). Available online at: https://www.who.int/data/nutrition/nlis/info/children-6-59-months-receiving-vitamin-a-supplements (accessed July 10, 2022).

[10] Imdad A Herzer K Mayo-Wilson E Yakoob MY Bhutta ZA. Vitamin A supplementation for preventing morbidity and mortality in children from 6 months to 5 years of age. Cochrane Database Syst Rev. (2010). 17:CD008524. 10.1002/14651858.CD008524.pub2 21154399

[11] Imdad A Mayo-Wilson E Haykal MR Regan A Sidhu J Smith A. Vitamin A supplementation for preventing morbidity and mortality in children from six months to five years of age. Cochrane Database Syst Rev. (2022) 3:CD008524. 10.1002/14651858.CD008524.pub4 35294044 PMC8925277

[12] UNICEF. Vitamin A deficiency October, 2021. Available online at: https://data.unicef.org/topic/nutrition/vitamin-a-deficiency/ (accessed July 10, 2022).

[13] BANK TW. Vitamin A supplementation coverage rate (% of children ages 6-59 months) - Sub-Saharan Africa. (2018). Available online at: https://data.worldbank.org/indicator/SN.ITK.VITA.ZS?locations=ZG (accessed July 10, 2022).

[14] Berde A Bester P Kruger L. Vitamin A supplementation among children aged 6-59 months in 23 sub-Saharan African Countries. Eur J Public Health. (2018) 28(suppl_4):cky214.087. 10.1093/eurpub/cky214.087 30755287 PMC10260408

[15] Federal Ministry of Health. Ethiopia National Expanded Programme on Immunization, Comprehensive Multi-Year Plan 2011–2015. Addis Ababa: Federal Minisitry of Health Addis Ababa (2010).

[16] Federal Ministry of Health [Ethiopia]. National Nutrition Program. Addis Ababa: FMoH (2015).

[17] Gatobu S Horton S Kiflie Aleyamehu Y Abraham G Birhanu N Greig A. Delivering vitamin A supplements to children aged 6 to 59 months: comparing delivery through mass campaign and through routine health services in Ethiopia. Food Nutr Bull. (2017) 38:564–73. 10.1177/0379572117708657 28528554

[18] Laillou A Baye K Zelalem M Chitekwe S. Vitamin A supplementation and estimated number of averted child deaths in Ethiopia: 15 years in practice (2005-2019). Matern Child Nutr. (2021) 17:e13132. 10.1111/mcn.13132 33336556 PMC8189216

[19] Central Statistical Agency [Ethiopia]. Ethiopia Demography and Health Survey 2016. Addis Ababa: ICF CatDp (2017).

[20] Ethiopian Public Health Institute ICF. Ethiopia Mini Demographic and Health Survey 2019: Key Indicators. Rockville, MD: EPHI and ICF (2019).

[21] Ethiopia FMOH. Health sector transformation plan (HSTP): 2015/16–2019/20. Addis Ababa, Ethiopia (2015).

[22] Health EMo. Health Sector Transformation Plan II (HSTP II): 2020/21-2024/25 (2013 EFY-2017 EFY). Addis Ababa: MOH (2021).

[23] Raut MK JReddy J Bera D Warvadekar K. Enablers of vitamin a coverage among children under five years of age from multi-country analyses of global demographic and health surveys in selected LMIC and LIC countries in Africa and Asia: a random forest analysis. Int J Community Med Public Health. (2019) 6:395–411. 10.18203/2394-6040.ijcmph20185279

[24] Adamu MD Muhammad N. Assessment of vitamin A supplementation coverage and associated barriers in Sokoto State, Nigeria. Ann Nigerian Med. (2016) 10:16–23. 10.4103/0331-3131.189803

[25] Berde AS Bester P Kruger IM. Coverage and factors associated with vitamin A supplementation among children aged 6–59 months in twenty-three sub-Saharan African countries. Public Health Nutr. (2019) 22:1770–6. 10.1017/S1368980018004056 30755287 PMC10260408

[26] Kassa G Mesfin A Gebremedhin S. Uptake of routine vitamin A supplementation for children in Humbo district, southern Ethiopia: community-based cross-sectional study. BMC Public Health. (2020) 20:1500. 10.1186/s12889-020-09617-1 33008352 PMC7532605

[27] Aremu O Lawoko S Dalal K. Childhood vitamin A capsule supplementation coverage in Nigeria: a multilevel analysis of geographic and socioeconomic inequities. Sci World J. (2010) 10:452878. 10.1100/tsw.2010.188 20890579 PMC5763688

[28] Semba RD de Pee S Sun K Akhter N Bloem MW Raju VK. Coverage of vitamin A capsule programme in Bangladesh and risk factors associated with non-receipt of vitamin A. J Health Popul Nutr. (2010) 28:143–8. 10.3329/jhpn.v28i2.4884 20411677 PMC2980876

[29] Haile D Biadgilign S Azage M. Differentials in vitamin A supplementation among preschool-aged children in Ethiopia: evidence from the 2011 Ethiopian Demographic and Health Survey. Public Health. (2015) 129:748–54. 10.1016/j.puhe.2015.03.001 25982948

[30] Gilano G Hailegebreal S Seboka BT. Geographical variation and associated factors of vitamin A supplementation among 6–59-month children in Ethiopia. PLoS ONE. (2021) 16:e0261959. 10.1371/journal.pone.0261959 34972168 PMC8719719

[31] Snijders TAB. Multilevel analysis. In:Lovric M, editor. International Encyclopedia of Statistical Science. Berlin, Heidelberg: Springer Berlin Heidelberg (2021), p. 879–882. 10.1007/978-3-642-04898-2_387

[32] Worldometer. Ethiopia Population 2022. Available online at: https://www.worldometers.info/world-population/ethiopia-population/#:~:text=The%20current%20population%20of%20Ethiopia,the%20latest%20United%20Nations%20data (accessed September 2, 2022).

[33] The World Bank In Ethiopia. GDP per capita (current US$) – Ethiopia. World Bank (2022). Podcast. Available online at: https://data.worldbank.org/indicator/NY.GDP.PCAP.CD?locations=ET (accessed September 2, 2022).

[34] Federal Democratic Republic of Ethiopia Ministry of Health. Health Sector Development Program IV 2010/11 – 2014/15. FMoH: Addis Ababa (2010).

[35] Lucha TA Engida TA Mengistu AK. Assessing the potential determinants of national vitamin A supplementation among children aged 6–35 months in Ethiopia: further analysis of the 2019 Ethiopian Mini Demographic and Health Survey. BMC Pediatr. (2022) 22:439. 10.1186/s12887-022-03499-5 35864488 PMC9306167

[36] Demsash AW Chereka AA Kassie SY Donacho DO Ngusie HS Tegegne MD. Spatial distribution of vitamin A rich foods intake and associated factors among children aged 6–23 months in Ethiopia: spatial and multilevel analysis of 2019 Ethiopian mini demographic and health survey. BMC Nutrition. (2022) 8:77. 10.1186/s40795-022-00573-0 35953835 PMC9367059

[37] The World Bank. Vitamin A supplementation coverage rate (% of children ages 6-59 months) - Low income. (2018). Podcast. Available online at: https://data.worldbank.org/indicator/SN.ITK.VITA.ZS?locations=XM (accessed September 2, 2022).

[38] Semba RD de Pee S Sun K Bloem MW Raju VJ. Coverage of the national vitamin A supplementation program in Ethiopia. J Trop Pediatr. (2008) 54:141–4. 10.1093/tropej/fmm095 18304953

[39] Nigusse T Gebretsadik AJ. Vitamin A supplementation coverage and ocular signs among children Aged 6–59 months in Aleta Chuko Woreda, Sidama Zone, Southern Ethiopia. J *Nutr Metab*. (2021) 2021:8878703. 10.1155/2021/8878703 33981457 PMC8088346

[40] Birhanu BE Kebede DL Kahsay AB Belachew AB. Predictors of teenage pregnancy in Ethiopia: a multilevel analysis. BMC Public Health. (2019) 19:601. 10.1186/s12889-019-6845-7 31101101 PMC6525551

